# Efficacy of Intravascular Ultrasound–Based 3D Wiring Using the Tip Detection Method for CTO Intervention

**DOI:** 10.1016/j.jacasi.2023.03.003

**Published:** 2023-05-09

**Authors:** Satoshi Suzuki, Atsunori Okamura, Hiroyuki Nagai, Masato Ishikawa, Satoshi Kameda, Kota Tanaka, Heitaro Watanabe, Gaku Nakazawa, Yasushi Sakata, Ichiro Shiojima

**Affiliations:** a Cardiovascular Center, Sakurabashi Watanabe Hospital, Osaka, Japan; b Department of Cardiology, Fujita Health University, Aichi, Japan; c Division of Cardiology, Department of Cardiovascular Medicine, Osaka University Graduate School of Medicine, Osaka, Japan; d Division of Cardiology, Kindai University Faculty of Medicine, Osaka, Japan; e Department of Medicine II, Kansai Medical University, Osaka, Japan

**Keywords:** chronic total occlusion, coronary intervention, IVUS-based 3D wiring, tip detection method

## Abstract

To perform intravascular ultrasound (IVUS)–based real-time 3-dimensional wiring in chronic total occlusion percutaneous coronary intervention, we devised a tip detection method and developed AnteOwl WR (AO)-IVUS, which is an upgraded version of Navifocus WR (Navi)-IVUS with an added pull back transducer system. We compared the procedural outcomes of AO-IVUS–based 3-dimensional wiring using the tip detection method (n = 30) and Navi-IVUS–based conventional wiring (n = 17) in chronic total occlusion percutaneous coronary intervention. The success rate of IVUS-guided wiring was markedly improved in the AO-IVUS group compared with the Navi-IVUS group (93% vs 59% of cases, respectively; *P =* 0.007). In cases of successful IVUS-guided wiring, the IVUS-guided wiring time was markedly improved in the AO-IVUS group compared with the Navi-IVUS group (9 ± 8 minutes vs 24 ± 26 minutes, respectively; *P =* 0.001). There were 2 successful cases of tip detection-antegrade dissection and re-entry in the AO-IVUS group.

Over the past decade, we have tried to standardize intravascular ultrasound (IVUS)–guided wiring as the key to maximizing the accuracy of guidewire manipulation in chronic total occlusion (CTO) percutaneous coronary intervention (PCI). In 2012, we developed Navifocus WR (Navi)-IVUS, the first CTO-specific IVUS, with a small profile and a short distance from the tip to the transducer in collaboration with Terumo Corp.^1^ The success rate could likely not be greatly improved. However, observation of the movement of the guidewires in the CTO lesions indicated the importance of 3-dimensional (3D) manipulation of the guidewires. Therefore, in 2014, we devised the 3D imaging rule and established angiography-based 3D wiring.^2^ Subsequently, to perform 3D wiring in real time even with IVUS-guided wiring, we devised a tip detection (TD) method and developed AnteOwl WR (AO)-IVUS (Terumo Corp), which is an upgraded version of Navi-IVUS with an added pull back transducer system.^3^ As AO-IVUS was launched in Japan in 2019, AO-IVUS–based real-time 3D wiring using the TD method has achieved the high success rate.^4^^,^^5^ While performing this wiring, we first found in August 2021 that the TD method allows for antegrade dissection re-entry (ADR) because the wall between the subintima and the true lumen can be punctured at the intended part in an exactly vertical direction using the IVUS observation. We named this method “tip detection antegrade dissection re-entry (TD-ADR).”^6^

In the present study, we compared the procedural results of Navi-IVUS–based conventional wiring and AO-IVUS–based real-time 3D wiring using the TD method in CTO-PCI.

## Methods

### Study population

We compared the procedural outcomes for the first 2 years after approval of each IVUS catheter at Sakurabashi Watanabe Hospital (Osaka, Japan). Between August 2012 and July 2014 after Navi-IVUS was first launched, 17 CTO-PCI patients underwent Navi-IVUS–based conventional wiring (Navi-IVUS group) among 171 consecutive CTO-PCI cases (96% success rate; the first operator was A.O. in 71% of cases, S.K., H.N., T.Y., or A.S. in the others). Between October 2019 and September 2021 after the launch of AO-IVUS, 30 patients undergoing CTO-PCI were treated with AO-IVUS–based real-time wiring (AO-IVUS group) among 168 consecutive CTO-PCI cases (97% success rate; the first operator was A.O. in 80% of cases, S.S., M.I., H.N., or K.T. in the others). These 47 patients (Navi-IVUS group; n = 17; AO-IVUS group; n = 30) were retrospectively enrolled in the present study. The review board of our institution approved the study protocol. All patients provided written informed consent to participate in this clinical study. The study was approved by the Central Medical Ethics Committee of Sakurabashi Watanabe Hospital, Osaka, Japan (approval #20-40).

### Interventional procedure, including methodology of NAVI-IVUS– and AO-IVUS–based wiring in CTO-PCI

We have already reported the specifications of Navi-IVUS (short-tip IVUS)^1^ and AO-IVUS (short-tip and pull back IVUS).^3^ Among the 4 major CTO revascularization strategies—antegrade guidewire escalation (AWE), retrograde guidewire escalation (RWE), ADR using the Stingray system (Stingray-ADR, Boston Scientific),^4^ and IVUS-guided wiring—we performed AWE followed by IVUS-guided wiring as often as possible instead of parallel wiring while following the CTO algorithms for CTO crossing. Stingray-ADR was not performed in the period of Navi-IVUS because we started it in 2018.

In antegrade wiring, the tip shape of CTO stiff wires was a 1-mm curve at an angle of 45°. A Corsair microcatheter (Asahi Intecc Co, Ltd) was mainly used because of sufficient backup support for the guidewires. When the antegrade first guidewire was advanced to around the CTO exit but could not be passed through the CTO lesion, it was highly probable that the first guidewire was advanced into the subintimal space. We moved on to using IVUS-guided wiring as much as possible instead of parallel wiring. The Corsair microcatheter was advanced through the guidewire around the CTO exit to create a space for the IVUS catheter, and if the guidewire was a tapered wire, it was changed to a 0.014-inch moderately stiff CTO wire to obtain good support for advancing the IVUS catheter. Using a double-chamber catheter, the second guidewire was then advanced and the microcatheter was advanced through this second wire, and the IVUS catheter was advanced through the first guidewire. When the IVUS catheter could not be advanced after the Corsair’s bougie, balloon dilatation was performed with a small-diameter balloon.

We performed the following 2 steps: positional information of vascular structures, such as the intimal space and exit lumen, was transferred from the IVUS image to the angiographic image as we reported previously^4^; and IVUS-based wiring was performed to advance the second guidewire to the target. In the Navi-IVUS group, the image-delayed IVUS-guided wiring was performed. The IVUS transducer was placed just beyond the transition site between the intimal space and subintimal space. The second guidewire was advanced to the position that was assumed to correspond to the target (intraplaque, exit lumen of the CTO, and so on) on angiographic images. On the other hand, in the AO-IVUS group, AO-IVUS–based real-time 3D wiring was performed.^3^ The TD method was performed to accurately navigate the guidewire to the target. During guidewire navigation, the tip and its direction were always visualized by moving the IVUS transducer back and forth at the tip part to visually construct the 3D image of the guidewire inside the vessel. In addition, the guidewire tip was accurately guided to the target under direct visualization while the transducer was always advanced in accordance with the advancement of the guidewire tip. From August 2021, we also performed TD-ADR to pass the re-entry routes.

### Study definition

CTO was defined as totally occluded lesion with an estimated duration of at least 3 months with thrombolysis in myocardial infarction grade 0. The J-CTO (Multicenter CTO Registry of Japan) score was applied to grade the difficulty of CTO lesions. The angiographic classifications, such as collateral connection grade, CTO entry type, calcification, bending, and lesion length, were defined as reported previously.^7^ Procedural success was defined as when both the guidewire and balloon crossed the occluded lesion completely, successfully dilating the occluded artery, and achieving restoration of antegrade flow (Thrombolysis In Myocardial Infarction grade flow score 3) with <50% residual stenosis on final angiography. In-hospital major adverse cardiac and cerebrovascular events consisted of cardiac and noncardiac death, Q-wave myocardial infarction, non–Q-wave myocardial infarction, target vessel failure followed by emergent target vessel revascularization with PCI or coronary artery bypass grafting, and stroke. Myocardial infarction was defined as an increase in creatine kinase level to more than twice the normal upper limit. Complications during the procedure consisted of vessel perforations without tamponade by the guidewire at the CTO site and perforations leading to cardiac tamponade.

### Statistical analysis

Numerical data are expressed as the median (IQR), whereas categorical values are expressed as percentages. Continuous data were compared using the Mann-Whitney *U* test because of the nonparametric nature of the data. Frequency functions were assessed using the Fisher exact test and the chi-square test. In all analyses, *P* < 0.05 was taken to indicate statistical significance. All statistical analyses were performed using commercially available software, EZR (Saitama Medical Center, Jichi Medical University).

## Results

Table 1 presents a summary of patient and lesion characteristics. Compared with the AO-IVUS group, the Navi-IVUS group has more patients with a history of coronary artery bypass grafting (18% vs 0%), fewer smokers (12% vs 57%), and more patients treated with hemodialysis (18% vs 0%). Table 2 shows the outcomes of the procedures. The success rate of IVUS-guided wiring was markedly improved in the AO-IVUS group compared with the Navi-IVUS group (93% vs 59%, respectively; *P =* 0.007). In the Navi-IVUS group, before Navi-IVUS–based wiring, the primary AWE was performed in all cases, followed by the rescue RWE in 53% (n = 9 of 17). The success rate of Navi-IVUS based wiring was 59% (n = 10 of 17). In the AO-IVUS group, before AO-IVUS–based wiring, the primary AWE was performed in 83% (n = 25 of 30) followed by the rescue RWE in 8% (n = 2 of 25), and the primary RWE was performed in 17% (n = 5 of 30) followed by Stingray-ADR in 80% (n = 4 of 5). After the failure of Stingray-ADR (n = 4), we then succeeded using AO-IVUS–based wiring in all of the 4 cases, which included the 2 cases recanalized by TD-ADR. The success rate of AO-IVUS–based wiring was 93% (n = 28 of 30).

**Table 1:** Demographic and Angiographic Characteristics

	Navi-IVUS Group (n = 17)	AO-IVUS Group (n = 30)	*P* Value
Demographic characteristics			
Age, y	67 (61-73)	68 (55-72)	0.706
Male	15 (88)	28 (93)	0.613
Clinical presentation			
Asymptomatic	8 (47)	16 (53)	0.766
Stable angina	9 (53)	14 (47)	
History of CABG	3 (18)	0 (0)	0.042
Coronary risk factors			
Hypertension	15 (88)	26 (87)	1.000
Diabetes mellitus	7 (33)	10 (59)	0.753
Dyslipidemia	14 (82)	27 (90)	0.653
Smoker	2 (12)	16 (57)	0.004
PAD	3 (12)	2 (11)	1.000
eGFR (<45 mL/min/1.73 m^2^)	4 (24)	1 (3)	0.051
Hemodialysis	3 (18)	0 (0)	0.042
LVEF <35%	3 (18)	3 (10)	0.889
Angiographic characteristics			
Target vessel			
RCA	5 (29)	6 (20)	0.588
LAD	6 (35)	15 (50)	
LCX	6 (35)	9 (30)	
LMT	0 (0)	0 (0)	
CABG	0 (0)	0 (0)	1.000
Collateral filling grade			
CC 0	1 (6)	2 (7)	0.276
CC 1	9 (53)	22 (73)	
CC 2	7 (41)	6 (20)	
CTO entry types			
Blunt	10 (59)	21 (70)	0.528
Severe calcification	4 (24)	13 (43)	0.218
Bending (>45°)	5 (29)	8 (27)	1.000
Occluded Length (>20 mm)	14 (82)	20 (67)	0.321
Reattempted lesion	2 (12)	10 (33)	0.165
ISR	2 (12)	1 (3)	0.544
J-CTO score	2 (1-3)	3 (1-3)	0.223
0	3 (18)	1 (3)	
1	2 (12)	7 (23)	
2	7 (41)	6 (20)	
3	3 (18)	11 (37)	
4	1 (6)	3 (10)	
5	1 (6)	2 (7)	

Values are median (IQR) or n (%).

AO-IVUS = AnteOwl WR intravascular ultrasound; CABG = coronary artery bypass grafting; CC = collateral connection grade; CTO = chronic total occlusion; eGFR = estimated glomerular filtration rate; ISR = in-stent restenosis; J-CTO score = Japanese chronic total occlusion score; LAD = left anterior descending coronary artery; LCX = left circumflex coronary artery; LVEF = left ventricular ejection fraction; Navi-IVUS = Navifocus WR intravascular ultrasound; PAD = peripheral arterial disease; RCA = right coronary artery.

**Table 2:** Procedure Outcomes, MACCE, and Complications

Total Cases of IVUS-Guided Wiring	Navi-IVUS Group (n = 17)	AO-IVUS Group (n = 30)	*P* Value
Frequency of use of computed tomography information	9 (53)	18 (60)	0.647
Frequency of use of 8-F guide catheter for antegrade approach	16 (94)	26 (87)	0.640
Frequency of use of the parallel wire technique before IVUS guide wiring	5 (29)	0 (0)	0.004
Predilatation to advance IVUS catheter			
Corsair's bougie only	6 (35)	17 (57)	0.176
Addition of balloon dilatation with a small-diameter balloon	11 (65)	11 (37)	
Rotablation	0 (0)	2 (7)	
Microcatheter in IVUS-guided wiring			
Corsair	7 (41)	23 (77)	0.026
Finecross	10 (58)	7 (23)	0.002
IVUS-guided wiring time, min	27 (15-45)	8 (5-12)	0.002
Total procedure time, min	208 (156-266)	143 (110-180)	0.002
Fluoroscopic time, min	113 (80-13)	72 (60-98)	0.002
RAD, mGy	6,627 (5,109-8,217)	3,172 (2,040-3,930)	< 0.001
Contrast dose, mL	220 (200-300)	130 (111-160)	< 0.001
Success rate of IVUS-guided wiring	10 (59)	28 (93)	0.007
Success rate throughout the procedure	15 (88)	29 (98)	0.170
MACCE			
Cardiac death	0 (0)	0 (0)	1.000
Noncardiac death	0 (0)	0 (0)	1.000
QMI	0 (0)	0 (0)	1.000
Non-QMI	0 (0)	0 (0)	1.000
Emergent target vessel revascularization with PCI or CABG	0 (0)	0 (0)	1.000
Stroke	0 (0)	0 (0)	1.000
Complications during the procedure			
Vessel perforation by the guidewire	0 (0)	0 (0)	1.000
Cardiac tamponade	0 (0)	0 (0)	1.000
Successful cases of IVUS-guided wiring	10	28	
Crossing wire in IVUS guided wiring			
Confianza 9g	0 (0)	4 (14)	0.562
Confianza 12g	9 (90)	16 (57)	
Confianza 20g	1 (10)	6 (21)	
Others	0 (0)	2 (7)	
IVUS-guided wiring time, min	16 (7-25)	8 (5-9)	0.067

Values are n (%) or median (IQR).

IVUS = intravascular ultrasound; MACCE = major adverse cardiac and cerebrovascular event(s); PCI = percutaneous coronary intervention; QMI = Q-wave myocardial infarction; RAD = radiation absorbed dose; other abbreviations as in Table 1.

The total procedure time, fluoroscopic time, radiation absorbed dose, and amount of contrast were significantly reduced in the AO-IVUS group than the Navi-IUVS group. In cases of successful IVUS-guided wiring, the IVUS-guided wiring time tended to be improved in the AO-IVUS group compared with the Navi-IVUS group *(P =* 0.067). The crossing wires in IVUS-guided wiring were mainly Confianza family wires in both groups. There were no in-hospital major adverse cardiac and cerebrovascular events or complications during the procedure in either group.

## Discussion

AO-IVUS–based 3D wiring has made it possible to perform high-precision guidewire manipulation in CTO lesions. In addition, the risk of tip hardness has been reduced, resulting in the easier step-up of the guidewires when needed. Therefore, the success rate of AO-IVUS–based 3D wiring exceeded 90% in short IVUS-guided wiring time. The 2 failure cases were true bifurcation cases in which there was a risk of the side branch occlusion caused by hematoma. In one patient when rescue RWE was performed, it resulted in successful recanalization, and in the other patient when primary RWE was performed at a later date, it resulted in successful recanalization. The absorbed radiation dose and amount of contrast were significantly reduced in the AO-IVUS group compared with the Navi-IVUS group. The main reasons were that the cine-angiography had been replaced with fluoroscopy in most of the PCI procedures and the coronary artery images created by computed tomography had been able to be moved in conjunction with the movement of the angiography detector to allow it to assume the vessel course in the CTO lesions.

While performing AO-IVUS-based 3D wiring, we succeeded in the intentional creation of the re-entry with an exact vertical directional puncture using the TD method (TD-ADR) in 2 cases even after the failure of Stingray-ADR. Compared with Stingray-ADR, TD-ADR enables the puncture to be at a more proximal region with greater accuracy because the target is clearly visible on IVUS images.^5^ AO-IVUS–based 3D wiring using the TD method ensures accurate and reliable guidewire navigation not only through the intraplaque routes but also through re-entry in CTO-PCI.

### Study limitations

The present study was based on retrospective data analysis of a relatively small number of patients from a single center.

## Conclusions

AO-IVUS–based real-time 3D wiring using the TD method markedly increased the success rate of IVUS-guided wiring in CTO-PCI compared with conventional IVUS-guided wiring.

## Funding Support and Author Disclosures

Dr Okamura has received speaker fees from Terumo Corp (Tokyo, Japan). All other authors have reported that they have no relationships relevant to the contents of this paper to disclose.
